# POD analysis of flow over a backward-facing step forced by right-angle-shaped plasma actuator

**DOI:** 10.1186/s40064-016-2361-8

**Published:** 2016-06-21

**Authors:** Bin Wang, Huaxing Li

**Affiliations:** School of Aeronautics, Northwestern Polytechnical University, 127 West Youyi Road, Xi’an City, 710072 Shaanxi Province People’s Republic of China

**Keywords:** Separated flows, Reynolds stress, Right-angle-shaped plasma actuator, Proper orthogonal decomposition

## Abstract

**Purpose:**

This study aims to present flow control over the backward-facing step with specially designed right-angle-shaped plasma actuator and analyzed the influence of various scales of flow structures on the Reynolds stress through snapshot proper orthogonal decomposition (POD).

**Methods:**

2D particle image velocimetry measurements were conducted on region (*x*/*h* = 0–2.25) and reattachment zone in the *x*–*y* plane over the backward-facing step at a Reynolds number of *Re*_*h*_ = 27,766 (based on step height $h {= 40 \text{ mm }}$ and free stream velocity $U_{\infty } {= 11 \text{ m/s}})$. The separated shear layer was excited by specially designed right-angle-shaped plasma actuator under the normalized excitation frequency *St*_*h*_ ≈ 0.345 along the 45° direction. The spatial distribution of each Reynolds stress component was reconstructed using an increasing number of POD modes.

**Results:**

The POD analysis indicated that the flow dynamic downstream of the step was dominated by large-scale flow structures, which contributed to streamwise Reynolds stress and Reynolds shear stress. The intense Reynolds stress localized to a narrow strip within the shear layer was mainly affected by small-scale flow structures, which were responsible for the recovery of the Reynolds stress peak. With plasma excitation, a significant increase was obtained in the vertical Reynolds stress peak.

**Conclusions:**

Under the dimensionless frequencies *St*_*h*_ ≈ 0.345 and $St_{\theta } \approx 0.0183,$ which are based on the step height and momentum thickness, the effectiveness of the flow control forced by the plasma actuator along the 45° direction was ordinary. Only the vertical Reynolds stress was significantly affected.

## Background

The separated and reattached flows have been the research hot topic in the aerodynamics field, which are not only an important flow phenomenon, but also would be met frequently in diverse practical fluid engineering applications. In general, the occurrence of flow separation is due to suddenly change surface, a separated flow usually gets development for the surface discontinuity and reattaches some location of downstream, which can form a recirculation zone if surface structure is allowed (Kostas et al. 2002). Previous experiments on reattaching separated flow have demonstrated that large-scale structures play a key role in flow dynamic (Bhattacharjee et al. 1986). Actually, the occurrence of coherent structure is one of the most prominent features about turbulent flows (Kline et al. 1967), the significant turbulence details usually are hidden within coherent structures or vortex characterized by organized motions (Shah and Tachie 2009). It is generally accepted that a better cognition of coherent structures is the key to research turbulence (Kostas et al. 2005). An in-depth understanding of coherent structures would also provide the possibility of elaborating the physical dissipated mechanism of turbulent energy and its control (Shah and Tachie 2009). Moreover, it is believed that the Reynolds stress plays a crucial role in the turbulence flow, which can affect turbulent energy transfer over the wall bounded turbulence flow. The skin friction and the occurrence of flow separation have direct relationship to the Reynolds stress. Therefore, effective manipulation on Reynolds stress can result in modifying the viscous stress so as to improve the effectiveness of flow control. However, there is a relative little of present understanding of the inter-relationship between Reynolds stress and various scales flow structures in the turbulent flow. If inter-relationship understanding can be improved, then it would be positive meaning for flow separation control and drag reduction.

Extracting and distinguishing coherent structures from turbulence flow remain challenging (Kostas et al. 2005). Proper orthogonal decomposition (POD) is an effective statistical tool for extracting prominent features and identifying coherent structures. POD was first applied by Lumley (1967) to extract coherent structures within turbulent flows; Sirovich (1987) implemented POD method as the snapshot POD based on ergodic theory. POD is widely used in research on turbulence in aerodynamics. For example, Kostas et al. (2002, 2005) performed POD analysis on velocity and vorticity fields over the backward-facing step (BFS) at two Reynolds numbers (*Re* = 580 and 4660). The large-scale flow structures are associated with the existence of $\left\langle {u^{\prime } u^{\prime } } \right\rangle$ and $- \left\langle {u^{{\prime }} v^{{\prime }} } \right\rangle$ for the downstream zone of the reattachment, and the fine-scale structure governed the vertical normal stress. Shah and Tachie (2009) conducted a POD analysis for turbulent flow over the transverse rib with various pressure gradients and systematically elaborated the influence of various scales of flow structures on Reynolds stress. The large-scale flow structures contributed to a greater extent on Reynolds shear stress than that on normal stress. Shi et al. (2010) investigated the wake characteristics of the 2D square cylinder affected by wall proximity through PIV measurements using POD. The results revealed the presence of vortex shedding, the relationship between changes in wake and influence of wall constraint, and energy transfer mechanism. Chiekh et al. (2013) presented the wake status excited by synthetic jet actuations and showed that the wake flow topology and energy distributions were changed by actuators. The POD analysis also revealed the actuation mechanism and the close relation between the influence of the actuator on the wake and the actuation phase. Lengani et al. (2014) investigated a laminar separation bubble through PIV measurements and POD analysis; vortex shedding induced by Kelvin–Helmholtz instability was identified by POD. The POD eigenvector was applied to sort and obtain phase-average measurement results. The deterministic characteristic was separated from the stochastic flow state by the PIV results. Shestakov et al. (2014) presented volumetric velocity measurements by PIV to identify the 3D flow organization of a slot jet. POD analysis was also conducted to extract coherent flow structures. The results indicated that the quasi-2D large-scale vortices are related to jet meandering, which can be aperiodically modulated in terms of amplitude. Moreover, the secondary longitudinal vortex roll played a key role in momentum transfer and flow mixing. Wu et al. (2015) studied the three-dimensional instantaneous topologies of large-scale turbulence structures in separated flow on the suction surface of the blade of a wind turbine during stall delay, analyzed the major contribution of these structures to the first two POD modes, and observed the statistical effects of large-scale and energetic structures on turbulence. The results showed that the peaks of some statistics were significantly reduced upon removal of turbulence structures from the flow.

BFS is a simple and commonly encountered geometric structure in the flow with separation and reattachment. Numerous studies explored flow control over BFS. Bhattacharjee et al. (1986) applied an acoustic speaker on the step to achieve the most effective flow control at *St*_*h*_ = 0.2 $({Re}_{h} = 32 \times 10^{ 3})$. Chun and Sung (1996) also conducted a flow control experiment over BFS by using an acoustic speaker located at the corner of the step; the optimum actuation was obtained at around *St*_*h*_ = 0.27 for ${Re}_{h}$ between 13 and ${33 \times 10}^{ 3}$. Roos and Kegelman (1986) fixed the oscillating flap at the step corner to reduce the reattachment length for the actuation at *St*_*h*_ of 0.2–0.3 $\left( {{Re}_{h} = 39 \times 10^{3} } \right)$. Yoshioka et al. (2001) applied a slit fixed upstream the separation point. The reattachment length was reduced by 30 % at *St*_*h*_ = 0.19 for ${Re}_{h}$  = 3700. Morioka and Honami (2004) applied a row of vortex generators to control the development of the reattachment flow over BFS. Stephen et al. (2010) constructed a step plasma actuator that can produce a jet flow along 45° direction; however, the influence on flow over BFS was not investigated. Majima et al. (2012) applied two synthetic jet actuators to influence the dynamic development of reattachment flow and vortex structure over BFS at low Reynolds number. A dielectric barrier discharge (DBD) plasma actuator fixed at the step vertical wall was applied by D’Adamo et al. (2014) to excite BFS flow in the laminar regime $\left( {{Re}_{h} = 1520} \right)$. The recirculation zone was reduced up to 37 %, and the results indicated that the optimum excitation frequency should be associated with Kelvin–Helmholtz instability. The recirculation bubble over the BFS flow was reduced using serpentine plasma actuator produced by Zare-Behtash et al. (2014). Zhang and Li (2014) used a synthetic jet to control the development of separated flow over BFS and analyze the influence of Reynolds stress on skin friction and flow separation. Koide et al. (2015) presented that separation flow over BFS was inhibited by plasma actuator modulated by pulsed wave and reattachment phenomenon. Pouryoussefi and Mirzaei (2015) applied a plasma actuator to investigate the influence of forcing frequency and various locations of actuator on the separation zone by measuring surface pressure over BFS. These findings suggest that the most effective forcing frequency is *St*_*h*_ = 0.27; the optimal location of the actuator was upstream of the step. Xu et al. (2015) presented flow control over BFS by using a new design of synthetic jet actuator and control mechanism for synthetic jet actuator. Benard et al. (2016) used a plasma actuator located at step corner to control separated flow over BFS. The optimization excitation state can be found by genetic algorithm, the experimental results indicated that the maximum reduction in reattachment length can be obtained by increasing periodicity perturbation under shear layer mode. Sujar-Garrido et al. (2015) also used a plasma actuator to control flow over BFS. Stereoscopic PIV was used to determine the influence of actuator position and forcing frequency on the reattachment location. The effect of the horizontal and perpendicular force on flow dynamics and the influence of modulation input signal on horizontal forcing were also investigated. The results indicated that the influence of plasma actuator produced along the horizontal direction jet on shear layer development is the optimal excitation state under excitation frequency $St_{\uptheta } \approx 0.011.$

This study mainly aims to analyze the influence of different scales of flow structures on Reynolds stress by snapshot POD method for flow over BFS with and without right-angle-shaped plasma actuator.

## Experimental setup

### Wind tunnel

**Fig. 1:**
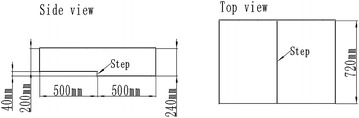
Parameters of test section

The experiments in this paper were conducted on the BFS flow control Comprehensive Experimental Platform mounted at the Northwestern Polytechnical University. The maximum experimental velocity of the test section is 20 m/s, and the minimum velocity is 7 m/s. The lowest turbulence intensity of free stream is 0.3 %. The upstream length of the step is 500 mm, the downstream length of the step is 500 mm. The step height *h* is 40 mm, and the width along the span is 720 mm. The expansion ratio (*ER*) is 1.2, and the aspect ratio (*AR*) is 10 (Fig. 1).

### Measurement system

**Fig. 2:**
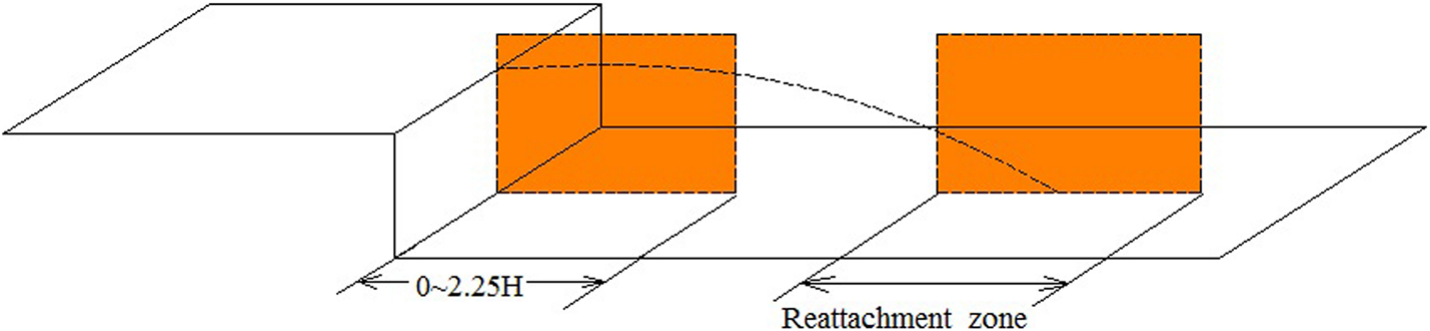
PIV measurement regions

2D PIV was applied in the experiment. The PIV system employed the Nd:YAG laser to generate a double-pulse-laser of 30 μs intervals at an acquisition frequency of 15 Hz. The amount of acquisition per experiment case is 200. The energy per pulse is less than or equal to 200 mJ, and the pulse width is 10 ns, the width of the laser is about 1 mm, and the maximum displacement of tracer particles in the *x*–*y* plane is about 4 pixels (double-pulse-laser of 30 μs intervals). The resolution of the CCD is 1600 pixels × 1200 pixels, and the field of view of PIV measurement is about 65 mm × 90 mm, the final spatial resolution is about a vector per 0.89 mm. The data post-process was operated with adaptive correlation by Dantec Dynamics studio software (the initial interrogation window size is 128 pixels × 128 pixels to the area of 32 pixels × 32 pixels with 50 % overlap). An error of ${\upvarepsilon }_{\text{u}} = 1.2\,\%$ (free stream velocity) was obtained in the PIV measurement of the instantaneous velocity field. Olive oil was sprayed into the flow to produce tracing particles with a diameter of about 1 μm for flow visualization. A laser sheet was oriented along the centerline of BFS. The field of view of PIV measurement was divided into two regions (Fig. 2) to improve spatial resolution over a large measurement field.

### Right-angle-shaped plasma actuator

**Fig. 3:**
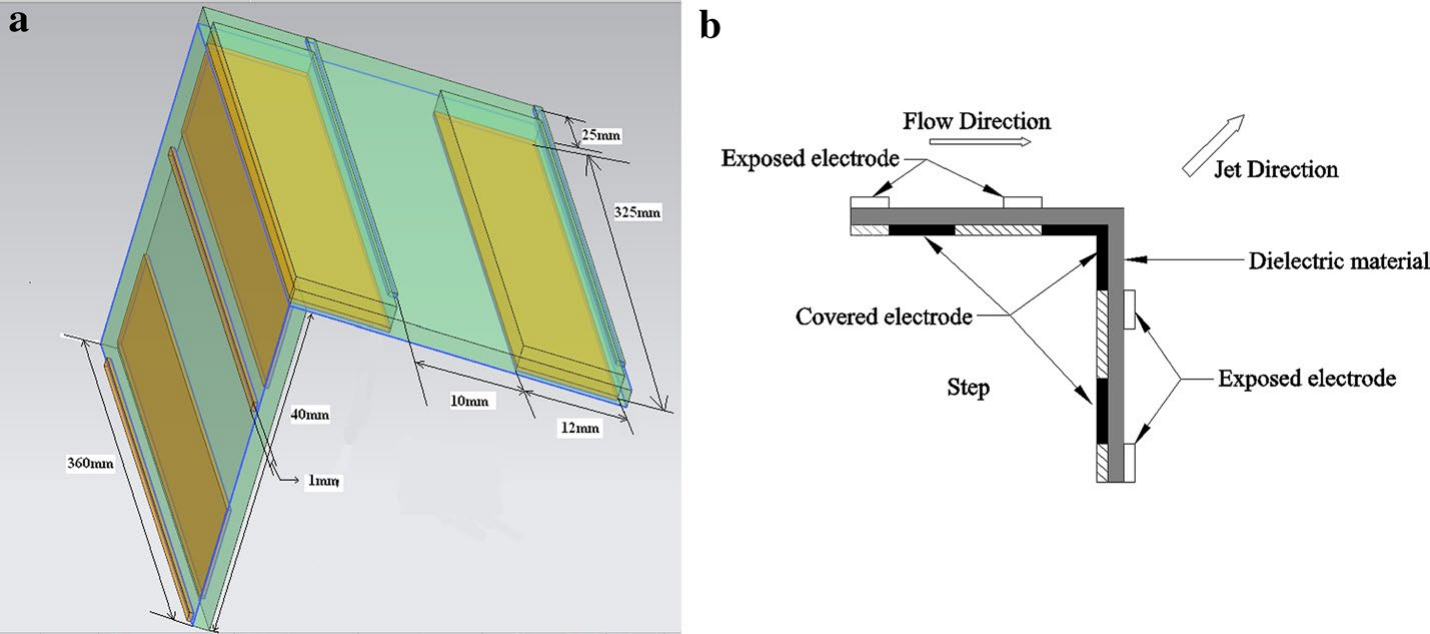
Schematic of right-angle-shaped plasma actuator. **a** 3D side view, **b** side view

The actuator was composed of two FR-5 printed circuit board [size: 40 mm (width) × 360 mm (length) × 2 mm (thickness), 38 mm (width) × 360 mm (length) × 2 mm (thickness)]. The electrodes composed of copper were installed on both sides of the circuit board. Figure 3b displays the structure of the specially designed plasma actuator for flow control on the BFS. Two pairs of electrodes are installed in the right-angle-shaped plasma actuator, with one pair placed along the horizontal plane and the other pair along the vertical plane. The width of the exposed electrodes was 1 mm, whereas the width of the covered electrodes was 12 mm, the offset distance between the closest ends of the two electrodes along the circuit board surface was 0 mm. A clearance of 25 mm was adopted for the exposed and covered electrodes, which were arranged at two side ends (spanwise direction) for wiring (Fig. 3a). The electrical parameters produced by plasma discharge were pulsed frequency of 95 Hz and input voltage of *Vpp* = 20.4 kV. The carrier frequency of *f*_ac_ = 1 kHz, and duty cycle was set as 50 %.

### Right-angle-shaped plasma actuator fixing

**Fig. 4:**
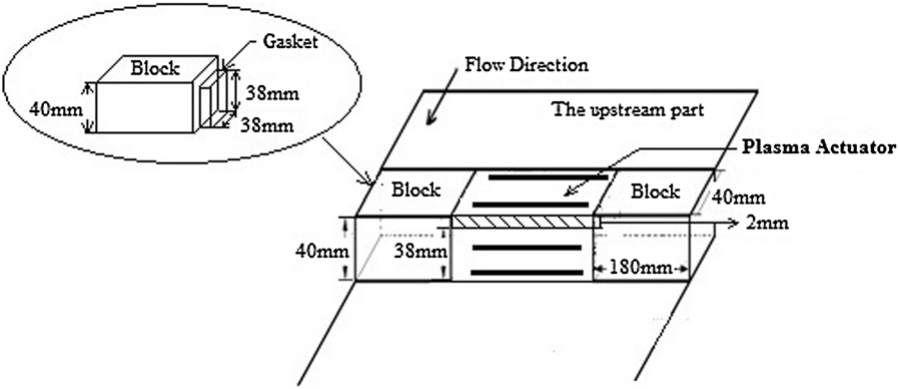
Fixing schematic of right-angle-shaped plasma actuator

The flow of the central cross-section was mainly observed during the experimental process. The right-angle-shaped plasma actuator should be installed at the central part of the BFS. Two hollow blocks were constructed (convenient for wiring) and placed at both sides of the test section. The right-angle-shaped plasma actuator was installed between the two blocks. The size of the block is shown in Fig. 4.

### Proper orthogonal decomposition (POD) basics

Proper orthogonal decomposition method has been introduced into the fluid mechanics field as a sophisticated mathematical method to objectively identify the dominant flow structure for given flow so far. According to the discrete dataset acquired by PIV, the snapshot POD method derived by Sirovich (1987) is applied to analyze the acquisition velocity field. The basic idea of snapshot POD is to obtain a set of optimal orthogonal eigenfunctions for a set of instantaneous velocity field (Berkooz et al. 1993; Zhou and Hitt 2004; Watanabe et al. 2015). In practical application, each instantaneous velocity field is regarded as a snapshot of the flow. The mean velocity field is calculated from a set of acquisition velocity field, the fluctuating velocity components can be obtained through subtracting the mean value from all instantaneous velocity field. The fluctuating velocity components are denoted by *u*, *v*, *w* respectively. All fluctuating velocity components for a set of instantaneous velocity field are arranged in a matrix U (Watanabe et al. 2015; Cavar and Meyer 2012; Shestakov et al. 2014).

The N × N autocovariance matrix is presented by ${\text{C}} = {\text{U}}^{\text{T}} {\text{U}}$. The next step, the corresponding eigenvalues problem can be solved through $\text{CA}= \uplambda \text{A},$ (A = A(t^n^) n = 1,…N). The eigenvector and eigenvalue can be obtained, as follows: ${\uplambda }^{ 1} { > \uplambda }^{ 2} { > } \cdots { > \uplambda }^{{{\text{N} - 1}}} { > \uplambda }^{\text{N}} .$. The normalized POD modes can be computed by equation below.

$${\Upphi}^{\text{i}} \left( {\text{x}} \right) = \frac{{\sum\nolimits_{\text{n = 1}}^{\text{N}} {{\text{A}}^{\text{i}} } \left( {{\text{t}}^{\text{n}} } \right){\text{u(x,t}}^{\text{n}} )}}{{\left\| {\sum\nolimits_{\text{n = 1}}^{\text{N}} {{\text{A}}^{\text{i}} } \left( {{\text{t}}^{\text{n}} } \right){\text{u(x,t}}^{\text{n}} )} \right\|}}\;\quad {{\rm i}} = \, 1 \ldots \, {{\rm N}}.$$

The expansion coefficients (POD coefficients) are determined by ${\text{a}}^{\text{n}} = {\uppsi }^{\text{T}} {\text{u}}^{\text{n}} ,\,{\uppsi} = \left[ {{\Upphi }^{ 1} { \Upphi }^{ 2} \ldots {\Upphi }^{\text{N}} } \right].$ So each instantaneous velocity fluctuating field can be reconstructed from the solved POD modes by ${\text{u}}^{\text{n}} = \sum\nolimits_{\text{i = 1}}^{\text{N}} {{\text{a}}_{\text{i}}^{\text{n}} {\Upphi }^{\text{i}} }$. The fluctuating energy corresponding POD mode can be presented by ${\text{E}}^{\text{k}} ={ \uplambda }^{\text{k}} / {\text{E}}^{\text{t}}$, where ${\text{E}}^{\text{t}} = \sum\nolimits_{\text{k = 1}}^{\text{N}} {{\uplambda }^{\text{k}} }$.

## Results and discussion

### Flow characteristics induced by right-angle-shaped plasma actuator

The natural instability frequencies at different streamwise positions over BFS were measured in advance by hot-wire anemometry and power spectra density (PSD) through fast Fourier transform method (FFT). The frequencies are 95, 70, 57, and 45 Hz at 0.5, 1, 2, and 3*h* step heights downstream of the step, respectively. Previous experiments by Bhattacharjee et al. (1986), Roos and Kegelman (1986), Chun and Sung (1996), Yoshioka et al. (2001), Pouryoussefi and Mirzaei (2015), and Sujar-Garrido et al. (2015) on BFS flow control reported that the optimal excitation frequency is close to the natural vortex shedding frequency. In this paper, the natural instability frequency (57 Hz) at the *x*/*h* = 2 streamwise location was scaled on the step height and momentum thickness. The dimensionless frequencies are *St*_*h*_ ≈ 0.21 and $St_{\theta } \approx 0.011,$ and the maximum reduction of the reattachment length was observed at this forcing frequency for the horizontal direction excitation. The experimental results are consistent with previously published results (Pouryoussefi and Mirzaei 2015, Sujar-Garrido et al. 2015). However, only a few studies investigated the subject at the natural instability frequency (95 Hz) at the *x*/*h* = 0.5 streamwise location (the dimensionless frequencies *St*_*h*_ ≈ 0.345 and $St_{\theta } \approx 0.0183$, which are based on the step height and momentum thickness), and the flow for regions *x*/*h* = 0–0.5 is closer to the free shear state. Furthermore, the flow was only slightly affected by recirculation zone. Thus, the pulsed frequency for the right-angle-shaped plasma actuator was modulated by the natural instability frequency of 95 Hz at the streamwise position of *x*/*h* = 0.5 of BFS. The influence of excitation with the jet along the 45° direction on flow dynamic evolution was observed in the restricted field of view.

**Fig. 5:**
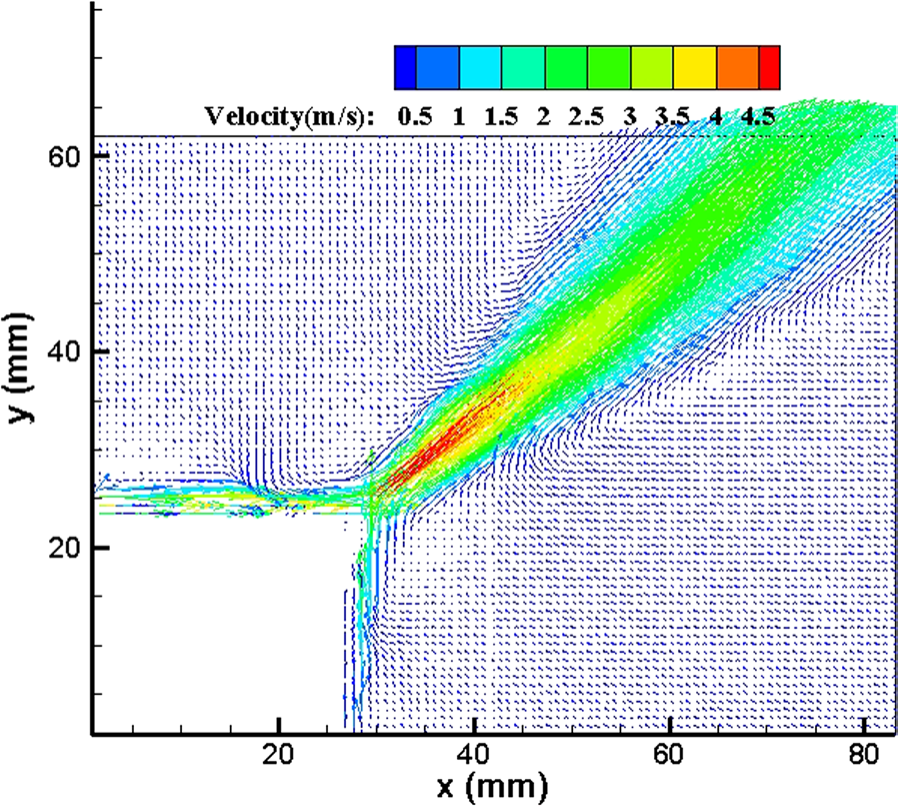
Mean velocity contour (*Vpp* = 20.4 kV)

**Fig. 6:**
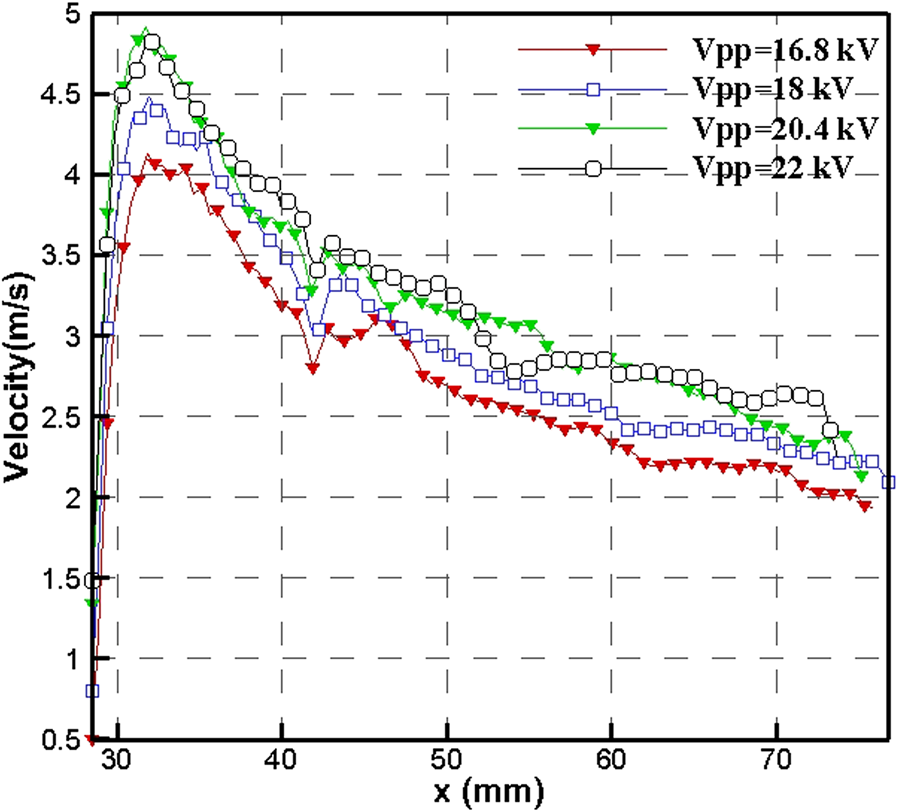
Velocity extracted along 45°

The induced velocity field by the plasma actuator was measured by PIV in the stationary air. We comprehensively investigated flow characteristics induced by the right-angle-shaped plasma actuator. Figure 5 shows the resultant velocity contour (time-averaged) without free stream measured by PIV under the pulsed frequency of 95 Hz and input voltage of *Vpp* = 20.4 kV. The induced velocity field exhibited a jet-like flow field with its central axis directed at a 45° radial line relative to the horizontal direction. The velocity gradually decayed as the distance away from the actuator along the 45° radial line increased; the jet flow field formed a slender triangular shape. Figure 6 shows the extracted resultant velocity along the 45° radial line under the pulsed frequency of 95 Hz with increasing input voltage from *Vpp* = 16.8 kV to *Vpp* = 22 kV. The resultant velocity peak value increased as input voltage augmented, but the resultant velocity did not reach the maximum value of 4.983 m/s at the maximum input voltage of 22 kV. The maximum resultant velocity of 4.983 m/s occurred at the input voltage of 20.4 kV. After the peak value, the velocity gradually decayed as the distance away from the actuator along the 45° radial line increased.

**Fig. 7:**
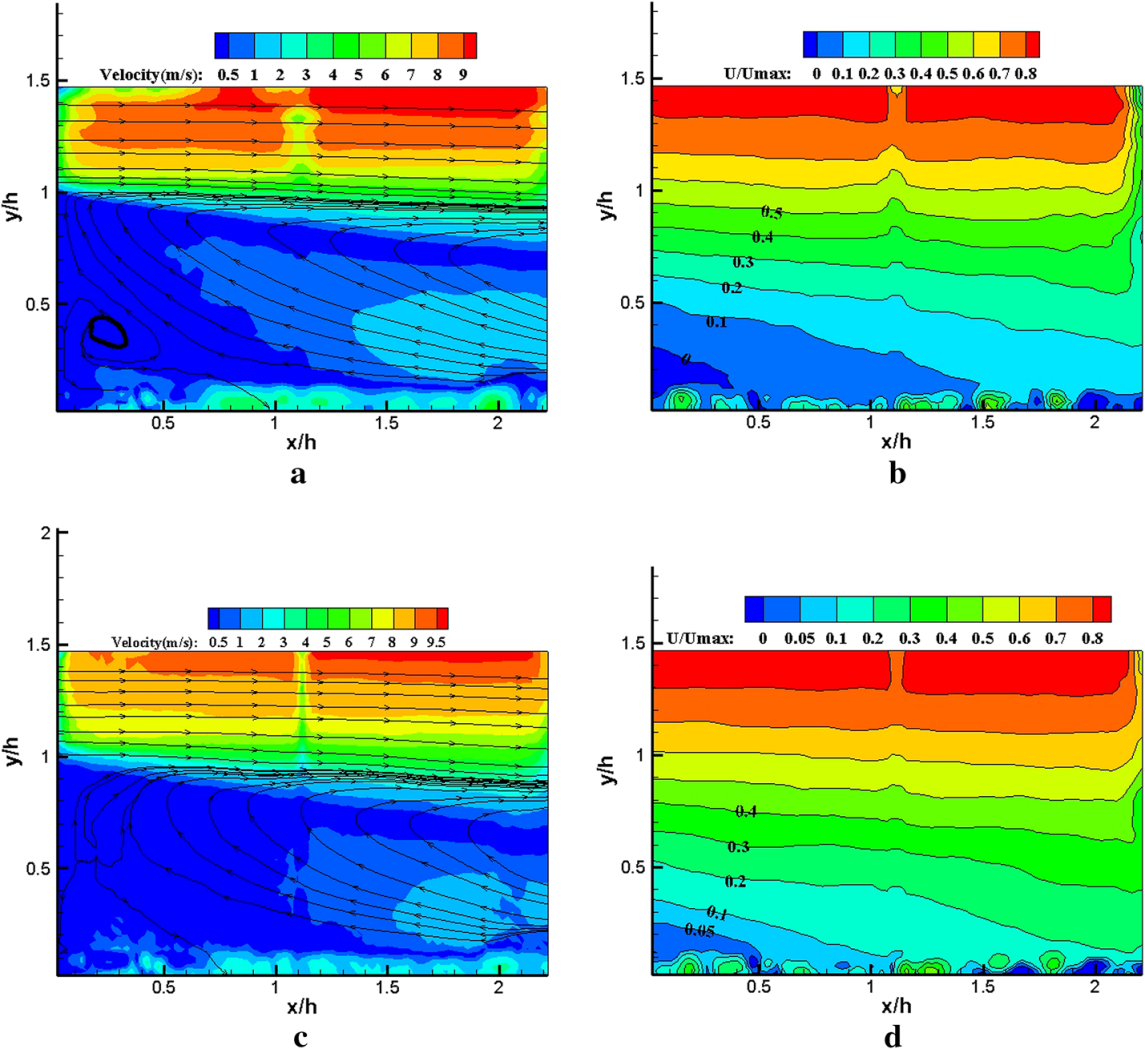
Mean streamlines and U/U_max_ = 0 m/s lines revealing reattachment location. **a** *x*/*h* = 0–2.25 zone (plasma off), **b** reattachment zone (plasma off), **c** *x*/*h* = 0–2.25 zone (*f* = 95 Hz, *Vpp* = 20.4 kV), **d** reattachment zone (*f* = 95 Hz, *Vpp* = 20.4 kV)

Figure 7 represents the time-averaged streamlines (*x*/*h* = 0–2.25 zone) and the *U*/*U*_*max*_ non-dimensional dividing line (reattachment zone) of the baseline and the actuation state (at *f* = 95 Hz, *Vpp* = 20.4 kV, i.e., *St*_*h*_ ≈ 0.345) for comparison. A partial circulation region without excitation is plotted in Fig. 7a, the height of main circulation region is approximately equal to the step height. We found a secondary vortex along the counter clockwise at the corner of the step. The reattachment point was approximately located at *x*/*h* = 0.5 in reattachment zone from Fig. 7b. However, for the excitation state (at *f* = 95 Hz and *Vpp* = 20.4 kV, i.e., *St*_*h*_ ≈ 0.345), the secondary vortex cannot be observed at the corner of the step (Fig. 7c). In the reattachment region, *U*/*U*_*max*_ = 0 non-dimensional dividing line cannot be found at the bottom of the step (Fig. 7d). The results indicate that the length of the recirculation region was reduced by excitation at *St*_*h*_ ≈ 0.345. This reduction of reattachment length could be attributed to the periodic forcing of excitation that affects the process of vortex pairing and amalgamation. Notably, the exposed electrode should influence the mean flow structure and reattachment location, but the influence may be extremely small. The influence of the exposed electrode on the mean flow and reattachment location was confirmed by Sujar-Garrido et al. (2015).

### Convergence and energy from dominant mode

**Fig. 8:**
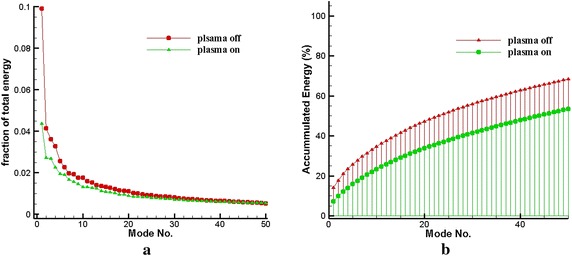
Spectra of turbulent kinetic energy: **a** Energy fraction of the first 50 modes, **b** accumulated turbulent kinetic energy $\sum {\uplambda }$%

The snapshot POD analysis may require a number of snapshots to determine the contribution of kinetic energy for a given mode, which is associated with the flow nature or complexity. If the number of snapshots is insufficient, this effect may result in inadequate convergence for the amount of energy in a mode (Kostas et al. 2005). Thus, energy convergence was analyzed to evaluate whether the amount of sample used in the current POD analysis is sufficient. POD analysis was carried out for the field of view ranging from *x*/*h* = 0–2.25 at the center-plane of BFS. With and without plasma excitation, the relative contribution of the first mode with increasing number of snapshots (${\uplambda }_{ 1} /\sum {\uplambda }_\text{n}$) for the region *x*/*h* = 0–2.25 was calculated from mode 1 to mode 200. The percentage of energy decreased with increasing number of snapshots (Table 1). For N > 150, the energy fraction is convergent, the energy fractions are ${\uplambda }_{ 1} /\sum {\uplambda_\text{n} = 10 \pm 0} . 1$ and ${\uplambda }_{ 1} /\sum {\uplambda_\text{n} = 4}{.5 \pm 0} . 1$ without and with plasma excitation, respectively, and no further modification exists. Therefore, N = 200 samples satisfied the convergence requirement in the present analysis. In Fig. 8a, plasma excitation was not employed, and the energy in the most dominant mode is 9.91 %. The number of modes that contained more than 1 % energy is 22, and the energy contained in each mode beyond the 22th mode is less than 1 %. The energy magnitude decreased by almost one order of magnitude compared with the first mode. Figure 8b shows that energy in the first 10 modes reached 33.13 %, and the energies associated with the first 22 and 50 modes are 48.19 and 67.85 %, respectively. We easily observed that nearly 50 % of energy is within the first 22 modes, whereas the energy within the first 10 modes (33.13 %) is more than the half of the energy in the first 22 modes. Hence, the first 10 modes would be associated with large-scale flow structures. Each mode beyond the 22nd mode contained relatively low energy, which means that the mode is associated with small-scale structures or background noise. With plasma excitation, energy in the first mode decreased to 4.36 %, with a decrease rate of 56 %. Energy in each mode beyond the 17th mode is less than 1 % (Fig. 8a). Energy contained in the first 10 modes was reduced to 21.9 % (Fig. 8b); energies associated with the first 17 and 50 modes are 30.03 and 52.87 %, respectively. Energy contained in various number modes significantly declined. In general, energy in each mode will exponentially decline with increasing number of modes (Fig. 8a). After the 50th mode, energy contained in each mode gradually slowed down and decayed (Fig. 8a). This difference of energy fraction may be related to the regular large-scale vortex motion caused by plasma excitation, resulting in the energy contained by the first mode was considerably decreased, and the energy distribution between the various modes would be more uniform.

**Table 1:** Accumulated turbulent kinetic energy ratio of mode 1 $\uplambda_{1}{/}\sum \uplambda_\text{n} \%$

Snapshots N	Energy fraction $\uplambda_{1}{/}\sum \uplambda_\text{n} \%$	
	Plasma off	Plasma on
10	29.9	19.9
25	19.4	11.7
50	14.6	8.2
100	11.5	5.8
150	10.4	4.8
170	10.1	4.6
180	10	4.5
190	9.9	4.4
200	9.9	4.4

The Reynolds stress convergence is shown in Table 2. A typical streamwise location was chosen for evaluation, i.e., {x/h, y/h} = {0.5, 1}, where the velocity gradient presented a relatively high value in the shear layer. At N > 150, the Reynolds stress was convergent (Table 2), the two normal Reynolds stresses are $$\left\langle {{\varvec{u}}^{{\prime }} {\varvec{u}}^{{\prime }} } \right\rangle$$$$1.17 \pm 0.002$$ and $$\left\langle {{\varvec{v}}^{{\prime }} {\varvec{v}}^{{\prime }} } \right\rangle$$$$0.526 \pm 0.002$$, respectively. The Reynolds shear stress is equal to $$\left\langle {{\varvec{u}}^{{\prime }} {\varvec{v}}^{{\prime }} } \right\rangle$$$$0.45 \pm 0.001$$. At the same streamwise location, the Reynolds stress by PIV was compared with the measurement data by hot-wire, as shown in the last line in Table 2 (the hot-wire sample frequency was set as 2000 Hz, and the sample time was about 33 s). Although a slight difference was found in the measurement results, this difference may be related to random noise in the POD analysis because only 200 snapshots were captured. The current PIV measurement results substantially satisfied the experimental requirement in this paper. In general, the Reynolds stress measured by PIV can be used for qualitative analysis in this paper.

**Table 2:** Reynolds stress convergence by increasing the number of sample N (plasma off)

Snapshots N	$\left\langle {{{u}}^{{\prime }} {{u}}^{{\prime }} } \right\rangle / {{U}}_{\infty }^{ 2} { \times 100}$	$\left\langle {{{v}}^{{\prime }} {{v}}^{{\prime }} } \right\rangle / {{U}}_{\infty }^{ 2} { \times 100}$	${ - }\left\langle {{{u}}^{{\prime }} {{v}}^{{\prime }} } \right\rangle / {{U}}_{\infty }^{ 2} { \times 100}$
100	1.063	0.435	0.441
150	1.197	0.526	0.462
170	1.172	0.526	0.453
175	1.168	0.528	0.446
180	1.171	0.5287	0.45
190	1.173	0.5294	0.451
200	1.176	0.53	0.45
Hot-wire	1.14	0.5057	0.48188

### The analysis of POD mode

**Fig. 9:**
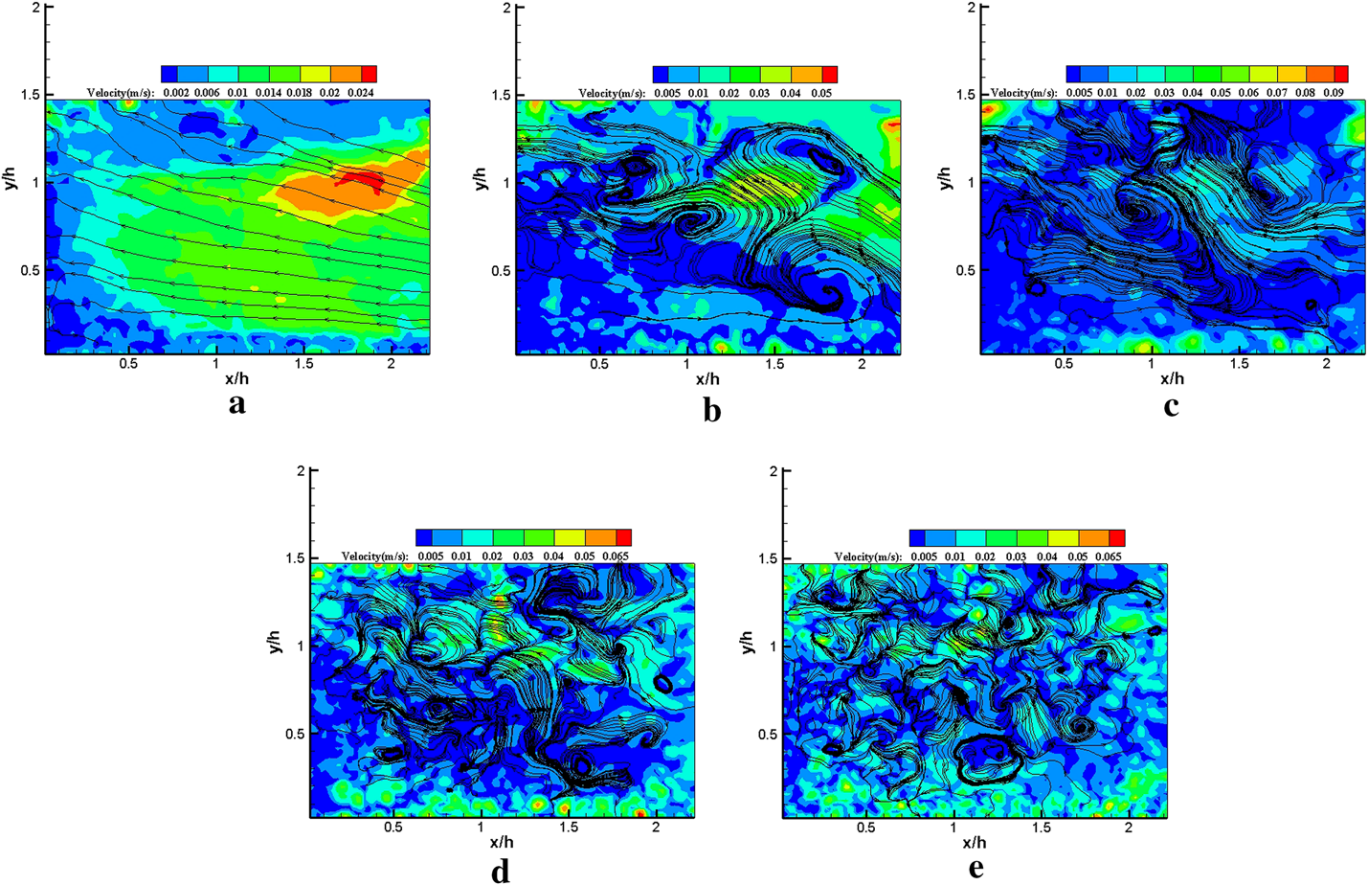
Streamline patterns **a** mode 1, **b** mode 5, **c** mode 10, **d** mode 50, **e** mode 100 (plasma off) from *x*/*h* = 0–2.25 region

**Fig. 10:**
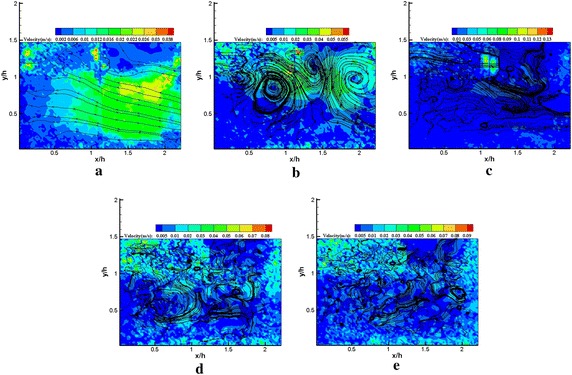
Streamline patterns **a** mode 1, **b** mode 5, **c** mode 10, **d** mode 50, **e** mode 100 (plasma on) from *x*/*h* = 0–2.25 region

The POD modes represent the flow state in the fluctuating field. Usually, the lower order modes would be associated with large-scale flow structures, which contained more energy. The higher order modes might be described by small-scale flow structures or noise. Thus, the lower and higher order modes were extracted to present some characteristics of various scale flow structures buried in the flow. Figures 9 and 10 represent the streamline patterns of various order modes with and without plasma excitation. In general, the number of vortex structures increases with the increasing order of the POD mode. Although rotating vortices were not observed in mode 1, the streamline patterns of the POD mode 1 are extremely similar with and without plasma excitation. A significant difference in vortex structures was found in mode 5 for excited and unexcited states. The regularized vortex street induced by the plasma actuator is displayed in Fig. 10b, which indicates the influence of plasma excitation on large-scale structures. Finding relatively large-scale flow structures with increasing order of the POD mode is difficult, instead the small scale flow structures gradually emerged; the effect of the plasma actuator on the small-scale flow structures was no longer observed. The streamline patterns of various order modes are consistent with the POD mode energy spectra. We emphasized the increase in random noise in the POD analysis from only 200 snapshots.

### Reconstruction analysis of Reynolds stress

**Fig. 11:**
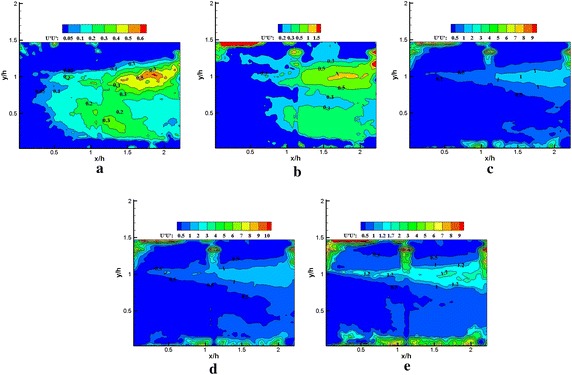
Normalized streamwise Reynolds stress $\left\langle {{{u}}^{{\prime }} {{u}}^{{\prime }} } \right\rangle / {{U}}_{\infty }^{ 2} \times 1 0 0$ obtained using **a** 1, **b** 1–5, **c** 1–25, **d** 1–50 modes in the reconstruction, **e** PIV data (plasma off)

**Fig. 12:**
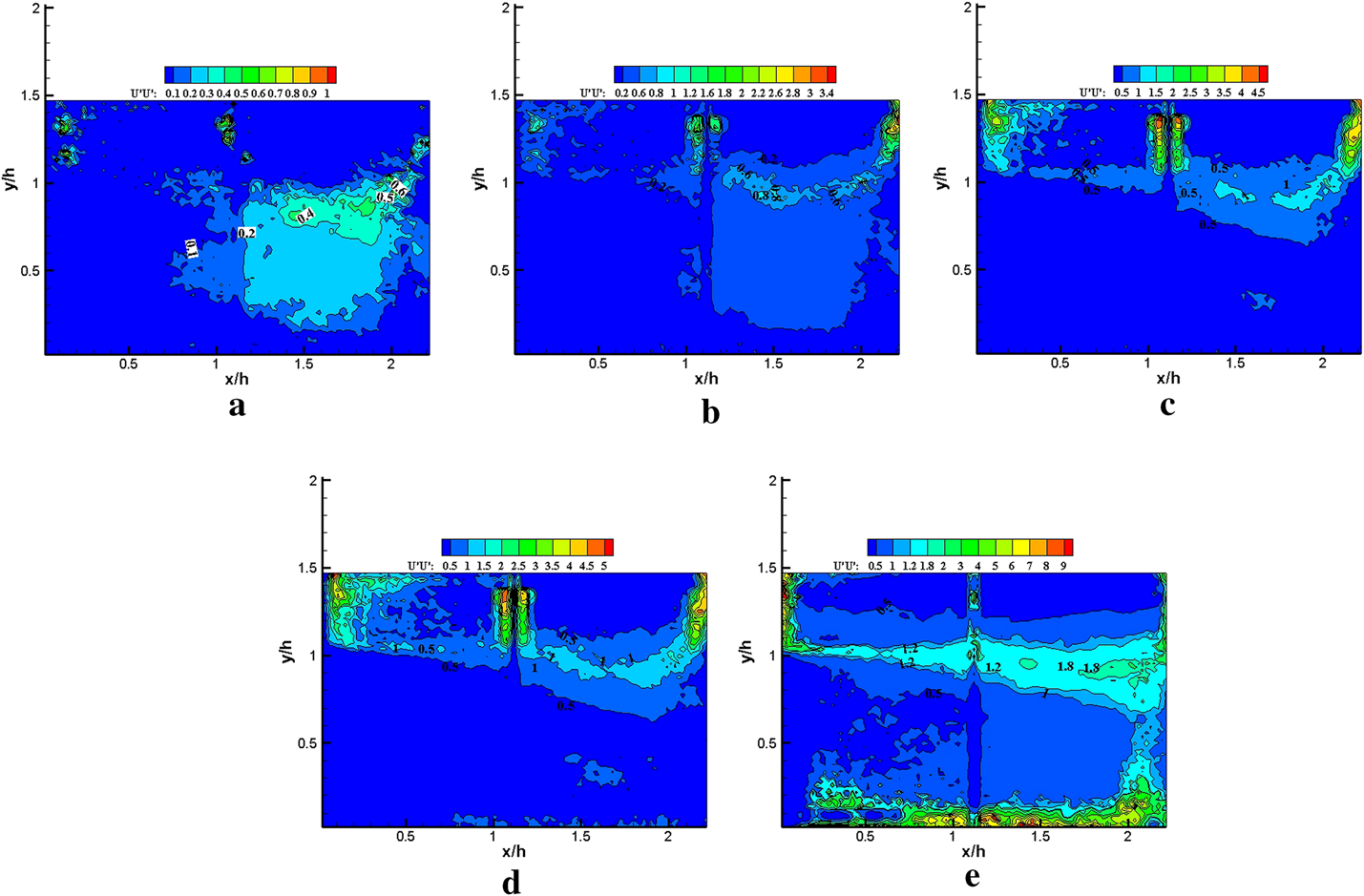
Normalized streamwise Reynolds stress $\left\langle {{{u}}^{{\prime }} {{u}}^{{\prime }} } \right\rangle / {{U}}_{\infty }^{ 2} \times 1 0 0$ obtained using **a** 1, **b** 1–5, **c** 1–25**, d** 1–50 modes in the reconstruction, **e** PIV data (plasma on)

**Fig. 13:**
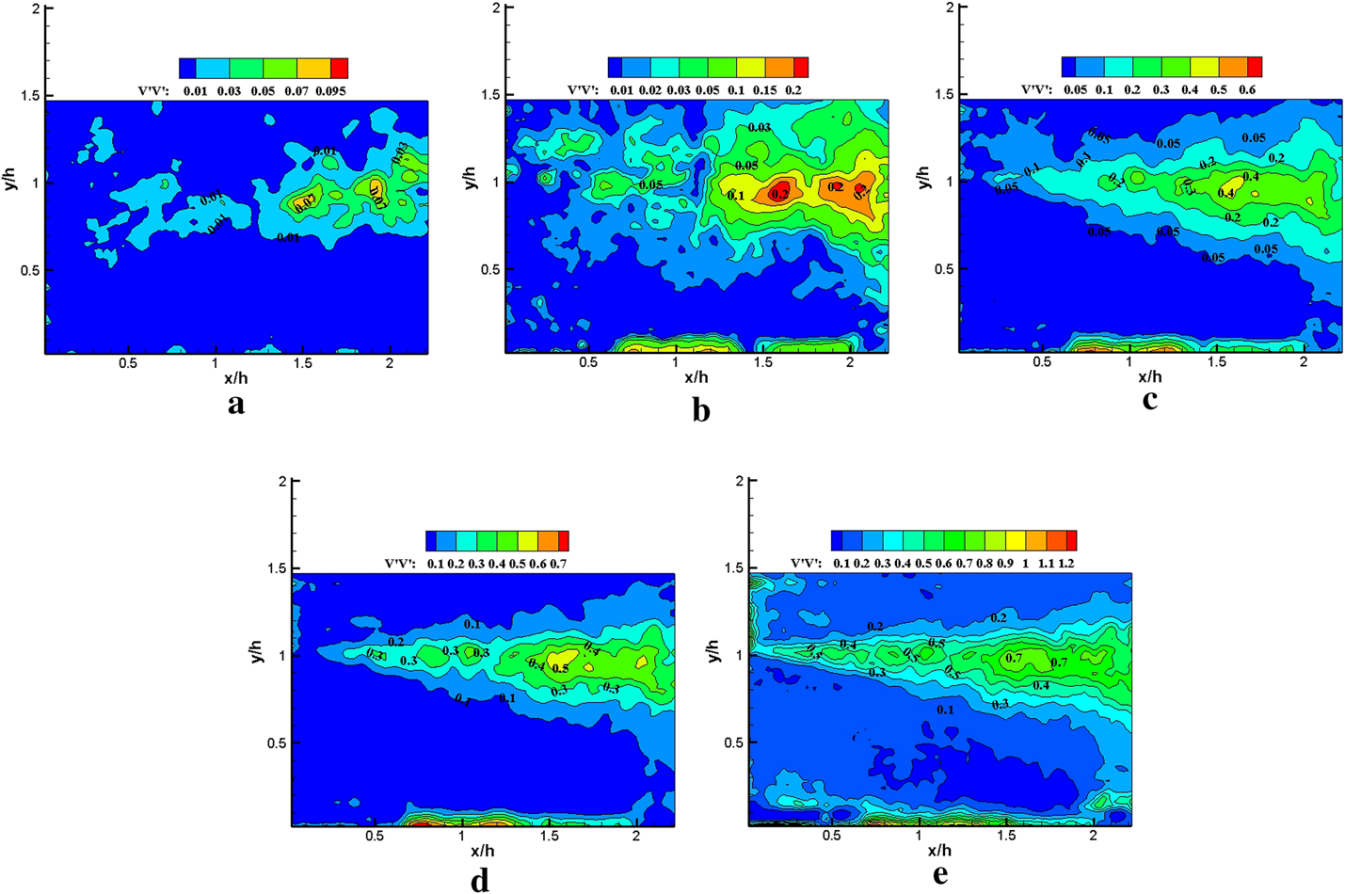
Normalized vertical Reynolds stress $\left\langle {{{v}}^{{\prime }} {{v}}^{{\prime }} } \right\rangle / {{U}}_{\infty }^{ 2} \times 1 0 0$ obtained using **a** 1, **b** 1–5, **c** 1–25, **d** 1–50 modes in the reconstruction, **e** PIV data (plasma off)

**Fig. 14:**
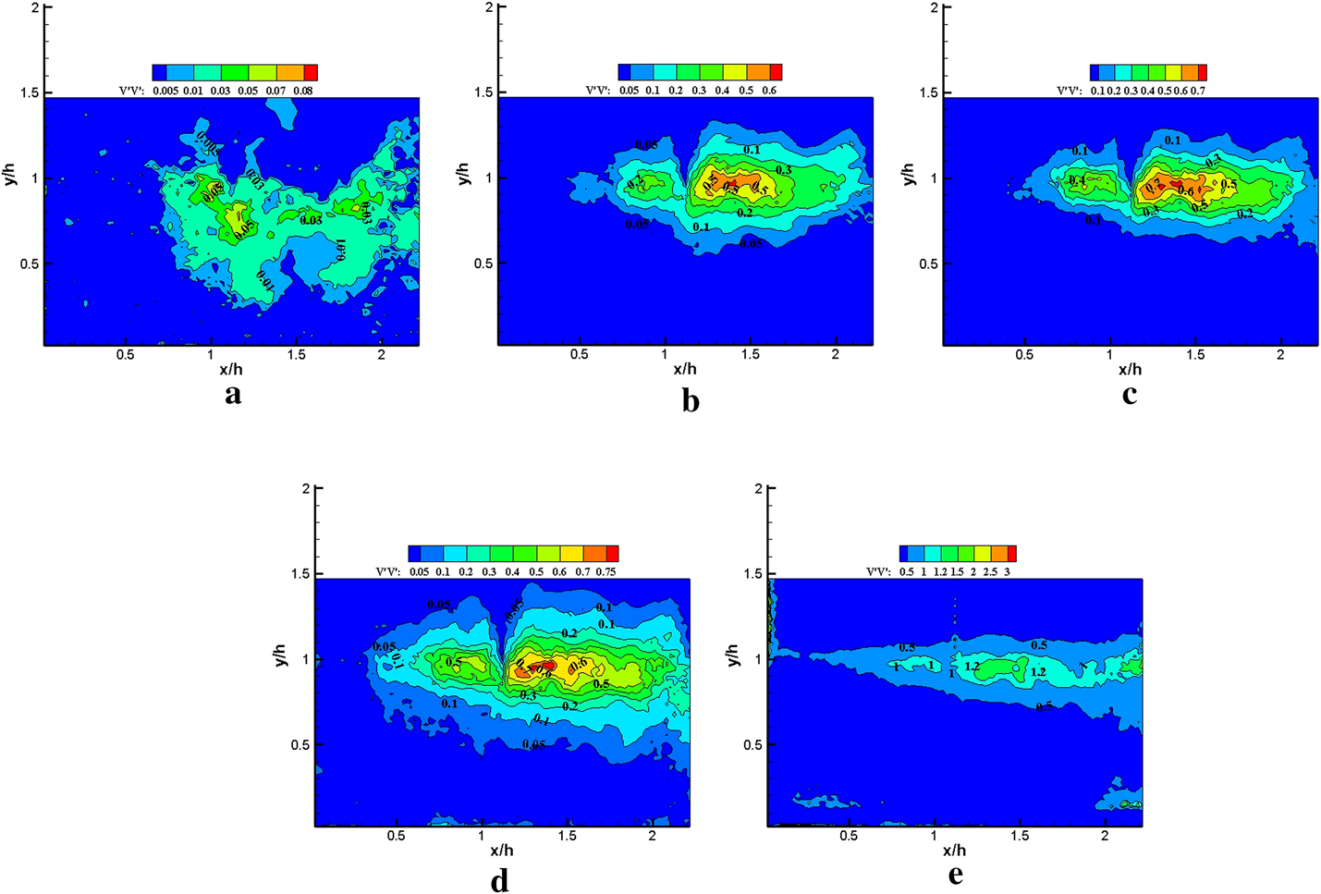
Normalized vertical Reynolds stress $\left\langle {{{v}}^{{\prime }} {{v}}^{{\prime }} } \right\rangle / {{U}}_{\infty }^{ 2} \times 1 0 0$ obtained using **a** 1, **b** 1–5, **c** 1–25, **d** 1–50 modes in the reconstruction**, e** PIV data (plasma on)

**Fig. 15:**
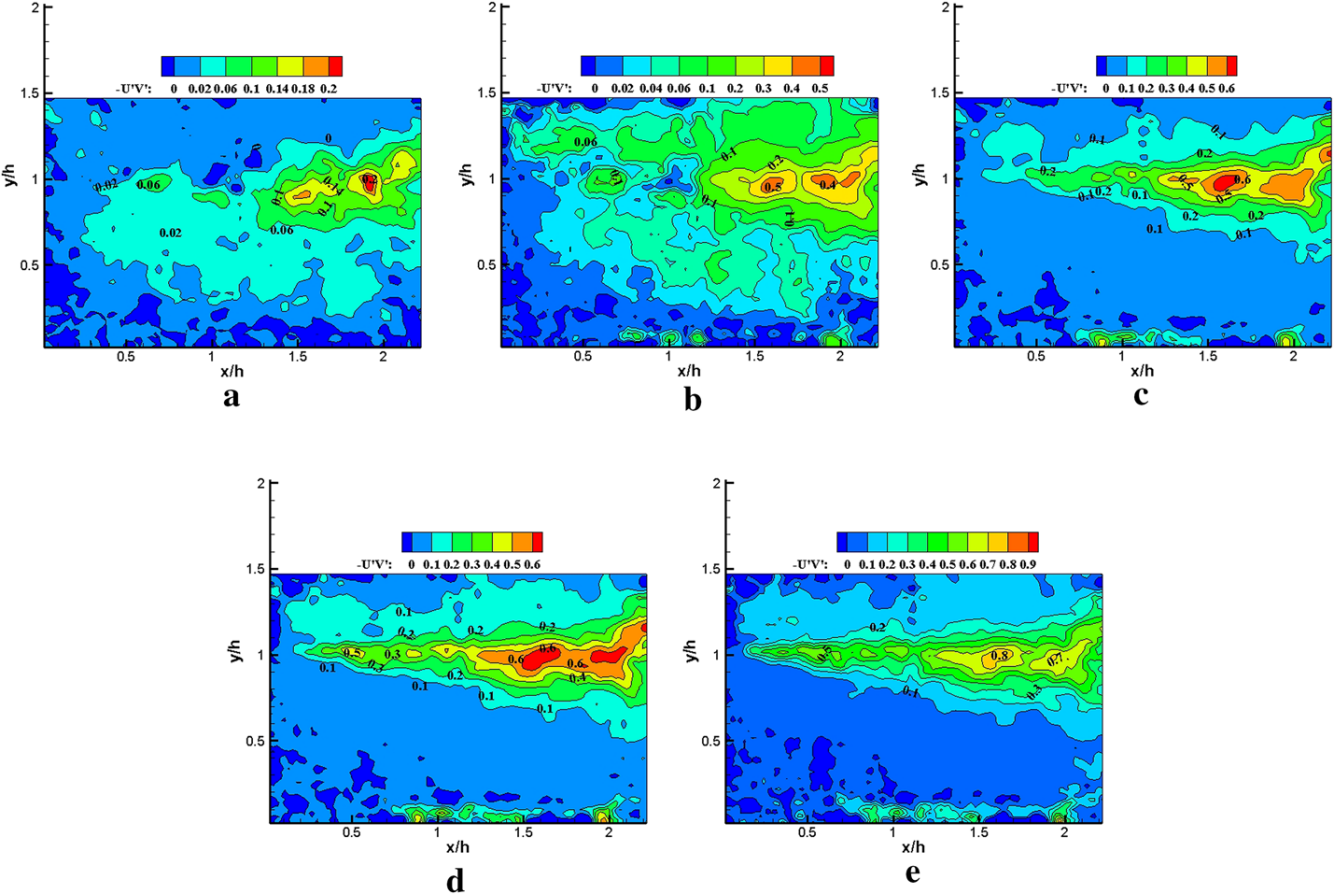
Normalized Reynolds shear stress $- \left\langle {{{u}}^{{\prime }} {{v}}^{{\prime }} } \right\rangle / {{U}}_{\infty }^{ 2} \times 1 0 0$ obtained using **a** 1, **b** 1–5, **c** 1–25, **d** 1–50 modes in the reconstruction, **e** PIV data (plasma off)

**Fig. 16:**
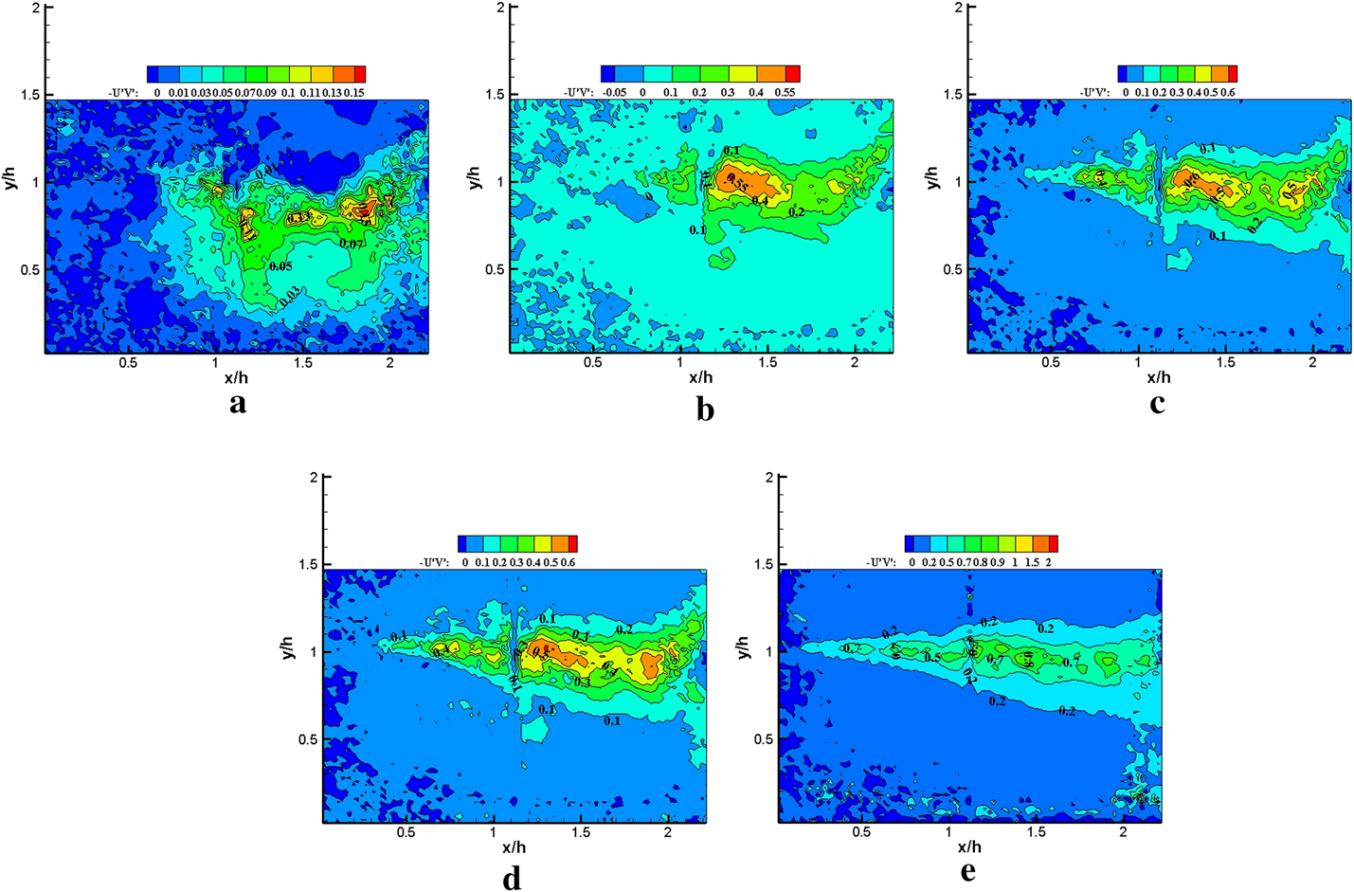
Normalized Reynolds shear stress ${ - }\left\langle {{{u}}^{{\prime }} {{v}}^{{\prime }} } \right\rangle / {{U}}_{\infty }^{ 2} \times 1 0 0$ obtained using **a** 1, **b** 1–5, **c** 1–25, **d** 1–50 modes in the reconstruction, **e** PIV data (plasma on)

The instantaneous velocity fields ${\text{u}}\left( {x, y, t_{n} } \right)$ for the region from *x*/*h* = 0 to *x*/*h* = 2.25 in the *x*–*y* plane were reconstructed using equation ${\text{u}}\left( {{x,y,t}_{n} } \right) \approx {U}\left( {x,y} \right){ + }\mathop \sum \nolimits_{\text{i = 1}}^{\text{N}} {\text{a}}_{\text{i}}^{\text{n}} {\Upphi }^{\text{i}}$. The reconstructions of the Reynolds stress spatial distribution were performed using POD mode 1, POD mode 1–5, POD mode 1–25, POD mode 1–50 in Figs. 11, 12, 13, 14, 15 and 16, respectively. The Reynolds stress spatial distribution by PIV measurements is also presented in Figs. 11, 12, 13, 14, 15 and 16 for comparison. Given the inhomogeneous spatial filtering property of POD (Adrian et al. 2000), we analyzed the influence of various scale flow structures on the mean turbulence structure through the cumulative effect of increasing the number of modes in reconstruction.

Figures 11, 12, 13, 14, 15 and 16 show that the spatial distribution of reconstructed Reynolds stress will gradually progress to approach PIV measurements with increasing number of modes in reconstruction. The energy in the relatively large region downstream of the step was captured by the first few lower order modes. This finding indicated that the flow development in that region was dominated by large-scale flow structures. Moreover, the peak Reynolds stress in the shear layer was not captured by the first few lower order modes. The small-scale flow structures appear to be responsible for the recovery of the peak of each Reynolds stress component.

In Fig. 11, the irregular streamwise Reynolds stress spatial distribution is significant to use in the first five modes in reconstruction without plasma excitation. This aspect may be related to sample size, resolution of the field of view (FOV), and random noise. When using an increasing number of modes in the reconstruction, the streamwise Reynolds stress spatial distribution localized in the narrow strip within the shear layer was gradually recovered. The results indicated that the higher order modes played a crucial role in recovering the streamwise Reynolds stress for the region centered in the shear layer. With plasma excitation (Fig. 12), the streamwise Reynolds stress was more slowly recovered than without plasma excitation. This result was particularly reflected within the region centered in the shear layer and implied that the influence of plasma excitation on the small-scale flow structures partially inhibited the recovery of the streamwise Reynolds stress. Moreover, no significant difference was noted in the PIV data between the excited and unexcited states (Figs. 11e, 12e).

In Fig. 13, more than 50 modes were required to recover the vertical Reynolds stress. The general spatial distribution of the vertical Reynolds stress in using the 50 modes in reconstruction qualitatively agrees with the PIV measurements without plasma excitation. In Fig. 14, the recovery of the vertical Reynolds stress was poorer than without plasma excitation, the spatial distribution and the peak of the vertical Reynolds stress were not fully recovered by using 50 modes in reconstruction with plasma excitation. However, the peak of vertical Reynolds stress is higher than that without plasma excitation for PIV data. This result indicates that the small-scale flow structures induced by the plasma actuator played a crucial role in improving vertical Reynolds stress.

In Fig. 15, the irregular Reynolds shear stress spatial distribution was also observed in using the first five modes in the reconstruction without plasma excitation. The reason may be similar to streamwise Reynolds stress. When an increasing number of modes were used in the reconstruction, the Reynolds shear stress spatial distribution achieved an effective reconstruction. Compared with the PIV data, the recovery of the Reynolds shear stress spatial distribution was achieved using 50 modes. Furthermore, more than 50 modes appeared to be required for the recovery of the Reynolds shear stress peak.

In Fig. 16, under plasma excitation, the spatial distribution of the reconstructed Reynolds shear stress through the five modes was not significantly modified. Increasing the number of modes in the reconstruction, the spatial distribution of the Reynolds shear stress was gradually recovered. The peak recovery of the Reynolds shear stress also required more than 50 modes. Moreover, for both excited and unexcited states, no significant change was observed in the peak of the Reynolds shear stress from PIV data. This result might be attributed to the occurrence of localized strong longitudinal shear produced by plasma actuator, which would imply that the vertical Reynolds stress would benefit from the energy redistribution by streamwise Reynolds stress. Thus, the spatial distribution of the highly intense Reynolds shear stress slightly changed, but the Reynolds shear stress peak was not modified.

The influence of the various scales flow structures on the spatial distribution of reconstructed Reynolds stress differed from one another. The relatively large-scale flow structures slightly contributed to the vertical Reynolds stress. This result can be observed in comparison between the spatial distribution of the reconstructed vertical Reynolds stress, reconstruction of Reynolds shear stress, and streamwise Reynolds stress distributions by using five modes. Only a very small portion distribution of vertical Reynolds stresses was recovered in comparison with the Reynolds shear stress and streamwise Reynolds stress. Furthermore, the influence of plasma excitation on the small-scale flow structures partially inhibited the recovery of streamwise Reynolds stress localized in the narrow strip within the shear layer. The peak of vertical Reynolds stress significantly increased with plasma excitation. However, no distinct modification was noted in the other two normal Reynolds stress.

## Conclusions

1. For the flow control over the BFS flow, the right-angle-shaped plasma actuator was specially designed, which can induce jet flow in a 45° direction. The jet resultant velocity reaches the maximum frequency of 4.983 m/s at *f* = 95 Hz and a voltage of *Vpp* = 20.4 kV.
2. The PIV measurements were carried out on the BFS flow with and without plasma excitation at *Re*_*h*_ = 27,766 (based on step height and free stream velocity 11 m/s). The pulsed frequency of the plasma actuator was modulated by 95 Hz. The dimensionless forced frequencies are *St*_*h*_ = 0.345 and $St_{\theta } = 0.0183$, respectively, based on the step height and momentum thickness. The mean reattachment point slightly moved to the upstream region. However, the optimum excitation frequencies (57 Hz) are *St*_*h*_ = 0.21 and $St_{\theta } = 0.011$, respectively, based on the step height and momentum thickness in this paper (not shown). The experimental results are consistent with a previously published conclusion (Bhattacharjee et al. 1986; Roos and Kegelman 1986; Chun and Sung 1996; Yoshioka et al. (2001); Pouryoussefi and Mirzaei 2015; Sujar-Garrido et al. 2015).
3. The POD analysis indicated that the relatively large-scale flow structures slightly contributed to vertical Reynolds stress, and the small-scale flow structures were involved in the recovery of the Reynolds stress peak in the region from *x*/*h* ≈ 1–2.25. With plasma excitation, the peak of the vertical Reynolds stress was significantly improved.
